# Acute Vision Loss as the Sole Presenting Symptom of Posterior Reversible Encephalopathy Syndrome

**DOI:** 10.7759/cureus.76042

**Published:** 2024-12-19

**Authors:** Noor H Ali, Reema E Alsulaiman, Masooma A Abbas, Fatema A Jamsheer, Njood Alsudairy

**Affiliations:** 1 General Practice, Eastern Health Cluster, Dammam, SAU; 2 General Practice, Almaarefa University, Diriyah, SAU; 3 General Practice, Salmaniya Medical Complex, Manama, BHR; 4 Radiology, Second Health Cluster, Jeddah, SAU

**Keywords:** computed tomography, hypertension, magnetic resonance imaging, pres, vision loss

## Abstract

A 45-year-old female with poorly controlled HTN presented with sudden, painless bilateral vision loss over 12 hours. On examination, she had only light perception in both eyes, with normal fundoscopy and no focal neurological deficits. Laboratory tests revealed mildly elevated creatinine and proteinuria. Imaging with MRI showed symmetrical hyperintensities in the occipital and parietal lobes, consistent with posterior reversible encephalopathy syndrome (PRES), while magnetic resonance angiography excluded large vessel occlusion. She was admitted to the intensive care unit, where blood pressure was controlled with intravenous labetalol, followed by oral antihypertensive therapy. Her vision improved within three days, and a repeat MRI on day five showed a resolution of the hyperintensities. The patient was discharged on day five with stable blood pressure and instructions for strict antihypertensive adherence. At a two-week follow-up, her visual acuity returned to baseline, and her serum creatinine normalized. This case emphasizes the importance of early diagnosis and management of PRES, particularly in patients with uncontrolled HTN, to prevent long-term neurological damage and recurrence.

## Introduction

Posterior reversible encephalopathy syndrome (PRES) is a clinical and radiological condition characterized by acute neurologic symptoms, including headache, confusion, seizures, and visual disturbances, typically associated with reversible white matter edema. PRES is often linked to hypertensive emergencies, though it can also be triggered by other factors, such as renal failure, immunosuppressive therapy, or eclampsia. The hallmark imaging findings of PRES are symmetrical hyperintensities in the posterior regions of the brain, particularly the occipital and parietal lobes [1,2].

The pathophysiology of PRES remains incompletely understood, but it is thought to result from an imbalance between cerebral perfusion pressure and blood-brain barrier integrity, leading to endothelial dysfunction and vasogenic edema. Severe, uncontrolled HTN is a major risk factor, as it can cause cerebral hyperperfusion and disrupt the blood-brain barrier, resulting in edema in the posterior brain regions [1-3]. While PRES can be associated with significant morbidity, it is considered reversible with prompt diagnosis and management, primarily focused on controlling blood pressure [2,4]. This case highlights a typical presentation of PRES in a middle-aged woman with poorly controlled HTN, emphasizing the importance of early recognition, imaging, and appropriate blood pressure management to prevent permanent neurological damage.

## Case presentation

A 45-year-old female presented to the ED with a sudden onset of painless vision loss in both eyes that had developed over the past 12 hours. The patient described the vision loss as a progressive blurring that eventually rendered her unable to perceive objects or recognize faces. She denied any preceding trauma, headache, or eye pain. Her medical history was significant for chronic HTN, which had been poorly controlled due to inconsistent medication adherence. She had no history of diabetes, hyperlipidemia, or prior cerebrovascular events. She was not on any regular medications apart from the occasional use of over-the-counter pain relievers and had no known allergies. Her family history was non-contributory, and she denied smoking, alcohol use, or illicit drug use.

Upon arrival, her vital signs were notable for elevated blood pressure of 190/110 mmHg. Other vital signs, including pulse rate, respiratory rate, and oxygen saturation, were within normal limits. On physical examination, the patient appeared alert and oriented, but she exhibited significant bilateral vision loss, with only light perception in both eyes. The pupils were equal, round, and reactive to light, with no relative afferent pupillary defect. Extraocular movements were intact, and the anterior segment and fundoscopy examinations did not reveal any signs of optic disc swelling, hemorrhage, or other abnormalities. Neurological examination was otherwise unremarkable, with intact cranial nerve function, normal motor strength, and no sensory deficits. No signs of meningismus or focal neurological findings were observed.

The initial diagnostic work-up included routine laboratory investigations, which revealed mildly elevated serum creatinine at 1.4 mg/dL (baseline unknown) and moderate proteinuria on urinalysis. Other laboratory parameters, including a complete blood count, liver function tests, and serum glucose, were within normal limits. A lipid panel showed borderline elevated low-density lipoprotein levels. Coagulation studies were unremarkable. Autoimmune and infectious workups were negative.

**Figure 1:**
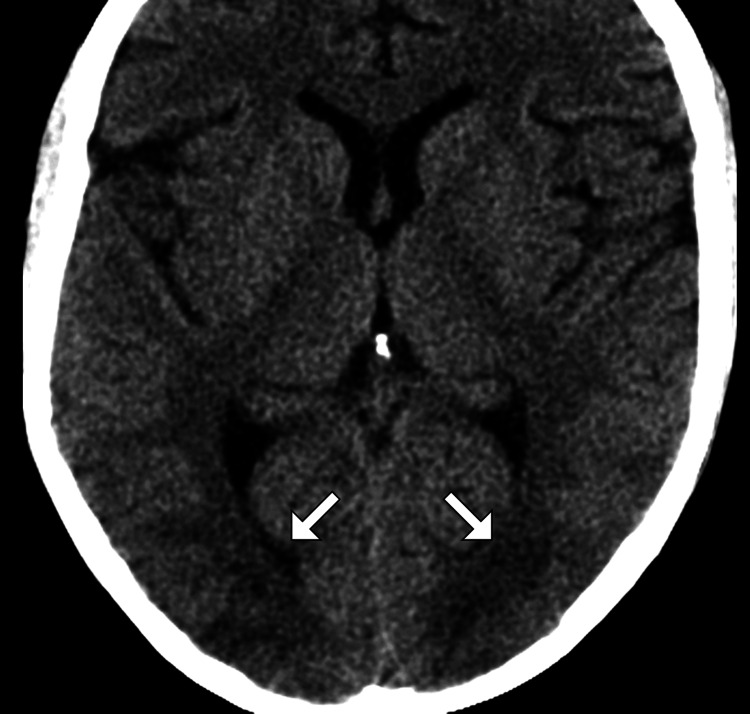
CT scan of the brain An axial image of the CT brain demonstrates bilateral hypodensity in the occipital lobes (arrow) with associated vasogenic edema. CT, Computed tomography

**Figure 2:**
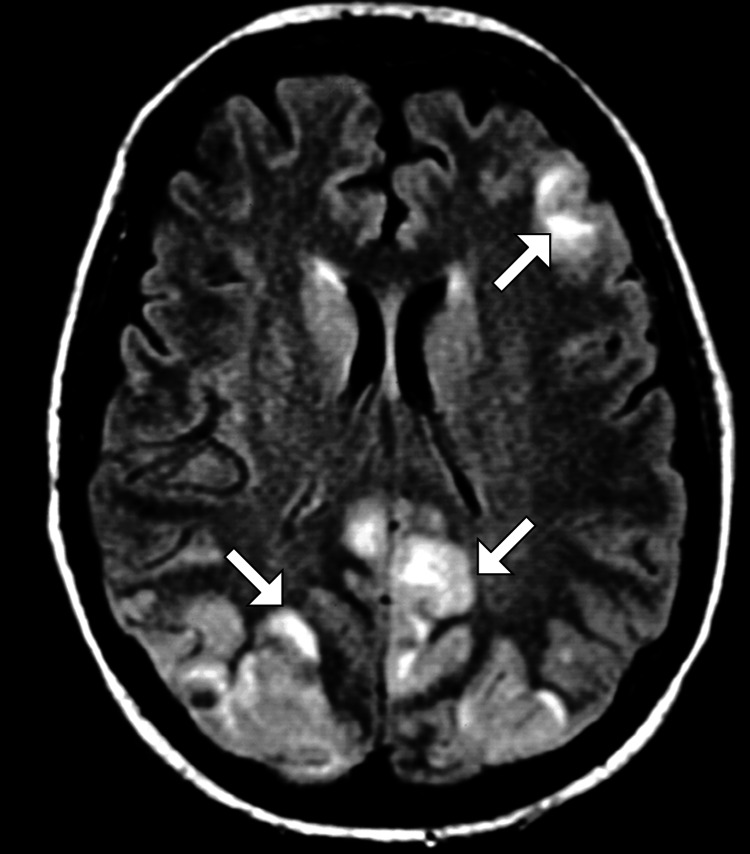
MRI of the brain An axial image of MRI of the brain demonstrates multifocal subcortical white matter with increased signal intensity (arrow) and associated vasogenic edema. MRI, Magnetic resonance imaging

Given the patient’s acute bilateral vision loss and severe HTN, imaging studies were promptly obtained. A non-contrast CT scan of the brain revealed no acute intracranial hemorrhage, mass effect, or ischemic changes (Figure 1). Subsequent MRI of the brain demonstrated symmetrical areas of hyperintensity on T2-weighted and fluid-attenuated inversion recovery sequences, predominantly in the occipital and parietal lobes, without evidence of restricted diffusion (Figure 2). These findings were highly suggestive of PRES. A magnetic resonance angiography excluded large vessel occlusion or significant vascular abnormalities. 

The primary differential diagnosis at this stage included hypertensive crisis with associated PRES, occipital lobe infarction, and toxic-metabolic encephalopathy. The absence of restricted diffusion on MRI and the lack of systemic toxic or metabolic derangements made the diagnosis of PRES most likely. The acute onset of symptoms in the setting of severe uncontrolled HTN further supported this diagnosis.

Management focused on the prompt reduction of blood pressure to target levels to prevent further complications. The patient was admitted to the ICU, where intravenous labetalol infusion was initiated for gradual blood pressure control. Blood pressure was titrated carefully to avoid cerebral hypoperfusion. Antihypertensive therapy was transitioned to oral agents, including amlodipine and losartan, as the patient stabilized. Supportive care included ensuring adequate hydration and monitoring renal function.

During her hospital stay, the patient showed gradual improvement in her visual symptoms. By the third day, she reported partial recovery of vision, with the ability to distinguish objects and recognize faces. A repeat MRI on day five showed significant resolution of the previously noted hyperintensities in the occipital and parietal lobes, consistent with the reversible nature of PRES. Blood pressure was well controlled with oral antihypertensive medications at discharge.

The patient was discharged home on the fifth hospital day with instructions to adhere strictly to her antihypertensive regimen and scheduled follow-up appointments. At her two-week outpatient follow-up, her visual acuity had returned to near baseline, and she reported no further episodes of vision loss. Repeat laboratory evaluations showed normalization of serum creatinine, and her blood pressure remained within the target range. She was counseled extensively on the importance of medication adherence and lifestyle modifications to mitigate the risk of recurrent hypertensive crises and PRES.

## Discussion

PRES is a neurotoxic disorder associated with acute-onset neurological symptoms, most commonly including headache, confusion, seizures, and visual disturbances, with imaging findings typically demonstrating reversible white matter edema [2-4]. This case of a 45-year-old female with sudden bilateral vision loss due to PRES emphasizes the critical role of early recognition and management, particularly in the context of uncontrolled HTN, which remains one of the most prevalent and significant risk factors for PRES. The patient’s presentation of progressive visual blurring that evolved into complete vision loss over 12 hours, along with her elevated blood pressure of 190/110 mmHg, strongly pointed toward a hypertensive crisis as the underlying etiology of PRES.

Hypertensive encephalopathy and PRES share similar clinical presentations, but the key differentiating factor is the reversibility of the symptoms with appropriate management [3,5]. Unlike hypertensive encephalopathy, which may present with generalized cerebral edema, PRES typically involves more focal areas of brain edema, often localized to the posterior regions, notably the occipital and parietal lobes, as seen on imaging. This pattern of involvement reflects the vascular anatomy and susceptibility of the posterior circulation to abrupt changes in blood pressure [2-4]. In this case, MRI imaging demonstrated symmetrical hyperintensities on T2-weighted and fluid-attenuated inversion recovery sequences, consistent with PRES, without evidence of restricted diffusion or acute ischemia, which ruled out an ischemic event and further supported the diagnosis.

One of the key diagnostic challenges in cases of PRES is distinguishing it from other conditions that may present with similar neurological symptoms. The differential diagnosis includes conditions such as occipital lobe infarction, toxic-metabolic encephalopathy, and retinal or optic nerve pathology [2,4]. The pathophysiology of PRES is thought to result from a failure in autoregulation of cerebral blood flow, leading to vasogenic edema. In the setting of severe HTN, as seen in this case, the disruption of the blood-brain barrier occurs due to endothelial dysfunction, particularly in the posterior cerebral circulation, which is more vulnerable to fluctuations in blood pressure. The posterior brain regions, especially the occipital lobe, are particularly prone to damage because of their dependence on the vertebrobasilar system, which is less able to compensate for rapid changes in blood pressure compared to the anterior circulation. As HTN is the most common risk factor for PRES, effective blood pressure management is crucial in preventing further cerebral damage.

In this case, prompt and aggressive management of HTN was paramount to the patient’s recovery. Intravenous labetalol was used to gradually lower the blood pressure, and blood pressure targets were carefully monitored to avoid cerebral hypoperfusion. The literature emphasizes that rapid reduction in blood pressure, while essential, must be done cautiously to prevent ischemic injury resulting from over-aggressive lowering. This approach is supported by guidelines, which recommend a stepwise approach to blood pressure reduction in PRES, with close monitoring of cerebral perfusion and organ function [2-5].

The patient’s visual disturbances gradually improved within three days, and repeat MRI on day five demonstrated resolution of the hyperintensities, highlighting the reversible nature of PRES when treated appropriately. This recovery is consistent with the prognosis in most PRES cases, as neurological symptoms tend to resolve with timely treatment of the underlying cause, such as blood pressure control in hypertensive cases. However, some studies have reported persistent neurological deficits, including cognitive impairment and visual disturbances, in a small proportion of patients, underscoring the importance of early diagnosis and management to prevent long-term sequelae [1-4].

Our case also underscores the importance of patient education and adherence to medication, as poor compliance with antihypertensive therapy was a significant factor contributing to the patient's hypertensive crisis and subsequent PRES. Studies have shown that the majority of PRES cases occur in patients with underlying uncontrolled HTN, and consistent adherence to antihypertensive therapy is critical in preventing recurrences of such events [2,5]. In this case, the patient was counseled on the importance of strict adherence to her prescribed antihypertensive regimen and follow-up appointments to reduce the risk of future complications.

## Conclusions

In conclusion, this case illustrates the importance of early recognition and appropriate management of PRES in patients with severe, uncontrolled HTN. Prompt imaging, particularly MRI, is crucial for diagnosing PRES and differentiating it from other potential causes of acute neurological symptoms. With timely and controlled blood pressure management, the prognosis for PRES is generally favorable, with most patients experiencing a complete resolution of symptoms. However, careful monitoring is necessary to avoid complications, and patient education regarding antihypertensive adherence is essential to prevent recurrence. This case reinforces the critical role of managing underlying HTN to mitigate the risk of PRES and other hypertensive emergencies.

## References

[1] Parasher A, Jhamb R (2020). Posterior reversible encephalopathy syndrome (PRES): presentation, diagnosis and treatment. Postgrad Med J.

[2] Ando Y, Ono Y, Sano A, Fujita N, Ono S (2022). Posterior reversible encephalopathy syndrome: a review of the literature. Intern Med.

[3] Fischer M, Schmutzhard E (2017). Posterior reversible encephalopathy syndrome. J Neurol.

[4] Gewirtz AN, Gao V, Parauda SC, Robbins MS (2021). Posterior reversible encephalopathy syndrome. Curr Pain Headache Rep.

[5] Racchiusa S, Mormina E, Ax A, Musumeci O, Longo M, Granata F (2019). Posterior reversible encephalopathy syndrome (PRES) and infection: a systematic review of the literature. Neurol Sci.

